# Pressure-sensitive paint technique for surface pressure measurements in a low-density wind tunnel

**DOI:** 10.1007/s12650-014-0239-9

**Published:** 2014-10-19

**Authors:** M. Anyoji, D. Numata, H. Nagai, K. Asai

**Affiliations:** 1 Institute of Space and Astronautical Science, Japan Aerospace Exploration Agency, 6-1-1 Yoshinodai, Chuo-Ku, Sagamihara, 252-5210 Japan; 2 Department of Aerospace Engineering, Tohoku University, 6-6-1 Aoba, Aoba-Ku, Sendai, Japan

**Keywords:** Pressure-sensitive paint (PSP), Mars wind tunnel, Low-pressure condition, Temperature calibration

## Abstract

**Abstract:**

A low-density wind tunnel called the Mars wind tunnel, has been developed at Tohoku University that can produce a high subsonic flow at low pressures for aerodynamic measurements of low-Reynolds-number aircraft wings aimed at developing aircraft applicable to the atmosphere on the planet Mars. Accurate surface pressure measurements on the wing are essential for analysis of not only aerodynamic performance, including lift and drag, but also the flow fields around the wing. This paper presents a surface pressure measurement technique using pressure-sensitive paint (PSP) applicable for Mars wind tunnel tests under low-pressure conditions. The results show that a PSP composed of palladium tetra(pentafluorophenyl) porphyrin (PdTFPP) and poly[1-(trimethylsilyl)-propyne] [poly(TMSP)] exhibits a high-pressure sensitivity at pressures as low as 1 kPa, and the absolute values of the static pressures measured by the PSP accorded well with the values derived from static pressure sensors used as a reference. A calibration methodology for the non-uniform pressure sensitivity on the test model, including a temperature calibration, is also established. The PSP technique clearly demonstrated pressure sensitivity over a distinctive low-pressure region inside a leading edge separation bubble on a flat plate at low Reynolds numbers.

**Graphical Abstract:**

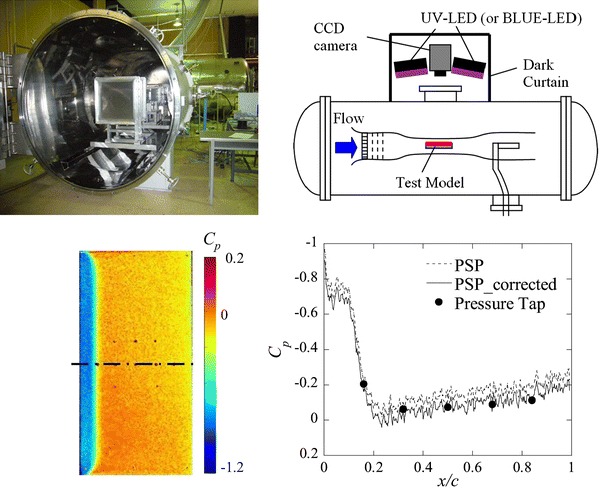

## Introduction

Aircraft applicable to the atmosphere on the planet Mars, or Mars airplanes, have been proposed and studied as a refreshing approach to explore the surface of the planet Mars (Guynn et al. 2003; Tanaka et al. 2005). Aircraft on Mars could cover a wide observation area and obtain unique scientific data from a few kilometers above the surface. However, conventional aircraft design criteria cannot be directly applied to the design of a Mars airplane because the Martian atmosphere is extremely different from that on the Earth. The atmospheric mean pressure is approximately only 0.7 kPa. Consequently, the flight condition of a Mars airplane is characterized by a low-Reynolds-number flow (*Re* = 10^3^ − 10^4^). The flow around the wings at such low Reynolds numbers easily separates, and a separation bubble forms after the separated shear layer reattaches to the wing surface. These separation characteristics cause nonlinear aerodynamic performance and disrupt the design of airfoils. Additionally, it is conceivable that a Mars airplane would fly at relatively high speed to produce sufficient lift to sustain its weight. Hence the effect of compressibility at low Reynolds number is also a critical element for the optimal design of the Mars airplane wing.

**Fig. 1:**
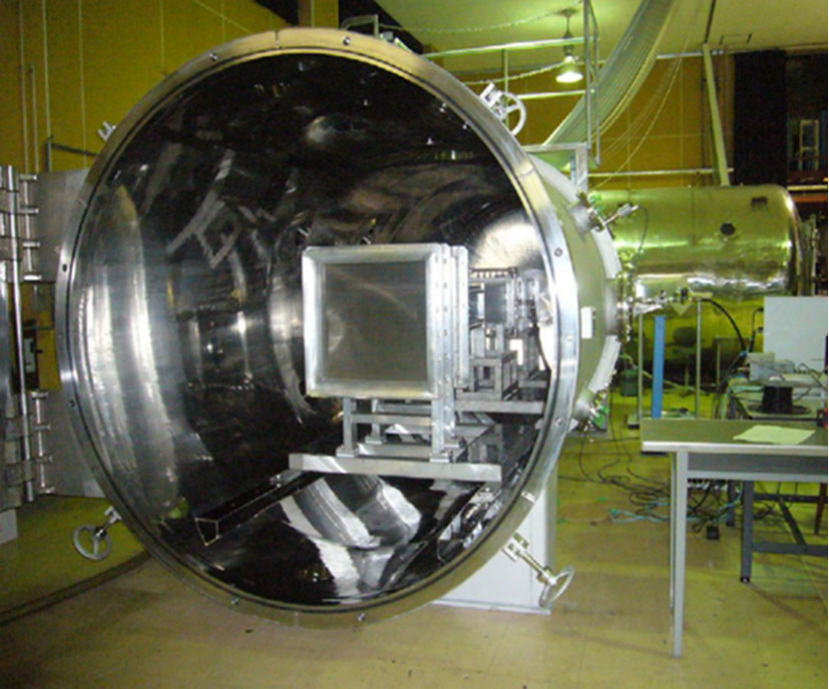
Mars wind tunnel (MWT) at Tohoku University

We have developed a low-density wind tunnel known as the Mars wind tunnel (MWT) at Tohoku University, as shown in Fig. 1, which is capable of simulating Martian atmospheric flight conditions, where a Reynolds number in the range from 10^3^ to 10^5^ and a Mach number higher than 0.7 can be attained (Anyoji et al. 2010, 2011). The most significant characteristic of this tunnel is that the Reynolds number effect and the Mach number effect on the aerodynamic performance can be analyzed independently.

Measuring the pressure distribution on airfoils under low-pressure conditions to understand the flow fields around airfoils is crucial because the separation characteristics, i.e., separation, transition, and reattachment, appear as a distinctive pressure-distribution pattern (Tani 1964; O’Meara and Mueller 1987; Gerakopulos 2010). However, it is well understood that under low-pressure conditions, a thin airfoil, which is difficult to equip with a conventional pressure sensor due to limitations of model construction, demonstrates higher aerodynamic performance than that of a thick airfoil (Oyama and Fujii 2006; Okamoto et al. 1996). In contrast, the pressure-sensitive paint (PSP) technique, which is a coated optical sensor, is applicable to thin airfoil models (Liu and Sullivan 2004).

The purpose of this study is to develop the PSP technique for quantitative surface-pressure distribution measurements in the MWT. The pressure distribution measurement using PSP is based on oxygen quenching of luminescent molecules. A typical PSP is composed of a luminescent molecule and a polymer binder which is permeable to oxygen. Poly[1-(trimethylsilyl)-propyne] [poly(TMSP)], which was first synthesized by Masuda et al. (1983), is a glassy material with a large free volume and has an extremely high oxygen permeability relative to all existing polymers. Mori et al. (2004) investigated the fundamental properties of three typical types of PSPs [platinum octaethylporphyrin (PtOEP), palladium tetra(pentafluorophenyl) porphyrin (PdTFPP), and platinumtetrakis (pentafluorophenyl) porphyrin (PtTFPP)] bound by poly(TMSP) in a pressure range below 14 kPa. The investigation results showed that PdTFPP exhibits high oxygen quenching and has the highest pressure sensitivity among the three PSPs. In this study, samples of PSP composed of PdTFPP and poly(TMSP) were tested using a calibration chamber and the fundamental properties of the PSP such as pressure sensitivity and photodegradation were evaluated at pressures less than 10 kPa. Furthermore, to demonstrate the applicability of the PSP for pressure measurements in the MWT, the pressure distribution on a blunt (flat-headed), flat-plate airfoil was measured in the Reynolds number range from 3.4 × 10^3^ to 3.2 × 10^4^, and the flow field around the airfoil was evaluated.

## Pressure-sensitive paint (PSP)

### Stern–Volmer equation

PSP is a coating-type sensor consisting of luminescent molecules and a binder. When illuminated with light of an appropriate wavelength, the luminescent molecules in the PSP are elevated to an excited state. The excited molecules return to the ground state through several photochemical mechanisms: luminescence, thermal deactivation, and oxygen quenching. The principle of PSP is based on oxygen quenching. In the presence of oxygen molecules, the energy of the excited molecules is transferred to the oxygen molecules and no luminescence is emitted. As a result, the luminescence intensity decreases with increasing oxygen concentration.

The luminescence quantum yield *ϕ* of PSP is expressed as the ratio of the luminescence intensity *I* to the absorption intensity *I*_a_ in accordance with the following equation (Liu 1997):

1 $$\phi = \frac{I}{{I_{\text{a}} }} = \frac{{k_{\text{L}} }}{{k_{\text{L}} + k_{\text{D}} + k_{\text{q}} [O_{2} ]}},$$

where *k*_L_ is the rate constant for the luminescence and *k*_D_ is the rate constant for the radiationless deactivation. The rate constant for oxygen quenching and the concentration of oxygen are expressed by *k*_q_ and [*O*_2_], respectively. The ratio of the luminescence intensity *I* to that in the absence of oxygen *I*_0_ is given by

2 $$\frac{{I_{0} }}{I} = \frac{{k_{\text{L}} + k_{\text{D}} + k_{\text{q}} [O_{2} ]}}{{k_{\text{L}} + k_{\text{D}} }} = 1 + \frac{{k_{\text{q}} [O_{2} ]}}{{k_{\text{L}} + k_{\text{D}} }} = 1 + k_{\text{q}} [O_{2} ]\tau_{0} ,$$

where *τ*_0_ = 1/(*k*_L_ + *k*_D_) is the luminescent lifetime in the absence of oxygen molecules. According to Henry’s law, the concentration of oxygen in a polymer binder is proportional to the partial pressure of oxygen $P_{{{\text{O}}_{2} }}$ or air pressure *P*:

3 $$[{\text{O}}_{2} ] = S(T) \cdot P_{{{\text{O}}_{2} }} = S(T) \cdot \phi_{{{\text{O}}_{2} }} \cdot P.$$

Here, *T* is the temperature, *S* is the oxygen solubility in the polymer binder layer, and $\phi_{{{\text{O}}_{2} }}$ is the mole fraction of oxygen in the test gas. For the present study, the mole fraction of oxygen $\phi_{{{\text{O}}_{2} }}$ is 21 %, and the oxygen solubility in the polymer binder layer *S* is a function of temperature. Hence, Eq. (2) can be put in the following form, known as the Stern–Volmer relation:

4 $$\frac{{I_{0} }}{I} = 1 + K_{\text{SV}} (T) \cdot P,$$

where *K*_SV_(*T*) is the Stern–Volmer constant.

As a practical issue, it is difficult to obtain *I*_0_ in the zero-oxygen condition. Instead of *I*_0_, a reference luminescent-intensity *I*_ref_ at a known reference pressure *P*_ref_ is typically used. As such, Eq. (4) is modified as

5 $$\frac{{I_{\text{ref}} }}{I} = A(T) + B(T)\frac{P}{{P_{\text{ref}} }},$$

where *A* (*T*) and *B* (*T*) are temperature-dependent calibration coefficients.

### Temperature dependence of luminescence intensity

The temperature dependence of the coefficients in Eq. (5) is attributed to the rate constant for the luminescence *k*_L_, the rate constant for the radiationless deactivation *k*_D_, and the rate constant for oxygen quenching *k*_q_. The luminescence intensity is significantly influenced by *k*_D_ and *k*_q_. The rate constant for radiationless deactivation *k*_D_ is expressed as the sum of a temperature-independent term *k*_0_ and a temperature-dependent term *k*_1_, as given in Eq. (6) below:

6 $$k_{\text{D}} = k_{0} + k_{1}$$

The rate constant *k*_1_ is given by the Arrhenius equation (Liu 1997) in Eq. (7).

7 $$k_{1} = C\;{ \exp }\left( {{{ - E_{\text{A}} } \mathord{\left/ {\vphantom {{ - E_{\text{A}} } {R_{0} T}}} \right. \kern-0pt} {R_{0} T}}} \right)$$

Here, *E*_A_ is the Arrhenius activation energy, *R*_0_ is the universal gas constant, and *C* is a pre-exponential factor. Equation (7) indicates that *k*_1_ increases with increasing temperature, and, thereby, *k*_D_ also increases with increasing temperature.

When a polymer is used as a binder, the rate for oxygen quenching *k*_q_ is dominated by the diffusion coefficient of the oxygen molecules *D*_Q_ in the polymer and given by the Smoluchowski relation (Liu 1997):

8 $$k_{\text{q}} = 4\pi N_{\text{m}} qD_{\text{Q}} ,$$

where *N*_m_ is the number of the molecules per millimole, and *q* is a factor that depends on the quenching mechanism.

The dependence of the diffusion coefficient of the oxygen molecules on temperature is also given by the Arrhenius relation (Liu 1997):

9 $$D_{\text{Q}} \propto \exp ( - E_{\text{Q}} /R_{0} T),$$

where *E*_Q_ is the activation energy for the diffusion of oxygen molecules in the polymer. Equation (9) indicates that *D*_Q_ increases with increasing temperature, indicating that *k*_q_ increases with increasing temperature, as shown in Eq. (8). Hence, it is apparent from Eq. (1) that the luminescence intensity *I* decreases with increasing temperature. Although the diffusion coefficient *D*_Q_ depends on the polymer employed as a binder, a larger *D*_*Q*_ results in a PSP with a higher pressure sensitivity. Therefore, it is considered that a binder with a larger *D*_Q_ will provide for better pressure sensitivity, particularly under low-pressure conditions (Niimi 2005).

### Temperature calibration

In typical wind-tunnel tests, the surface pressure can be calculated from the ratio of luminescence intensity images obtained at the wind-on and wind-off (reference) conditions. The temperature of the working fluid in the wind-on condition increases with time, and this temperature change causes a surface temperature-change on a test model. Because the luminescence intensity of the PSP changes with temperature, it is necessary to compensate for the effect of temperature change during the test. Brown (2000), Bell (2004), and Mitsuo et al. (2005) suggested that a raw image taken immediately after a tunnel shutdown be used as a wind-off image to minimize errors due to the temperature change. In addition, Yamashita (2007) proposed a temperature correction method based on a temperature-correction factor *α* (*T*). Using *α* (*T*), Eq. (5) can be transformed to the following expression:

10 $$\frac{{I(P_{\text{ref}} ,T_{\text{ref}} )}}{I(P,T)} = \alpha (T) \cdot \left( {A(T) + B(T)\frac{P}{{P_{\text{ref}} }}} \right),$$

11 $$\text{where} \,\;\,\alpha \left( T \right) = I\left( {P_{\text{ref}} ,T_{\text{ref}} } \right)/I\left( {P_{\text{ref}} ,T} \right)$$

and *T*_ref_ is the reference temperature.

In this method, the wind-off data obtained just after wind-tunnel shutdown are used so that the error caused by a spatial distribution of the model temperature can be minimized. In addition, *α* (*T*) is calculated using a single-point measurement of the model surface temperature under the following two specific conditions.

1. *A*_ref_ (*T*) and *B*_ref_ (*T*) which are Stern–Volmer coefficients for the case of *T* = *T*_ref_ are independent of temperature.
2. *α* (*T*) is uniform over the test model surface.

The PSP satisfying the first condition is denoted as the ideal PSP (Bell 2004; Gouterman et al. 2004), and, under these conditions, the Stern–Volmer relation [Eq. (4)] can be expressed by the following equation.

12 $$\frac{{I(P_{\text{ref}} ,T_{\text{ref}} )}}{I(P,T)} = \alpha (T) \cdot \left[ {A_{\text{ref}} + B_{\text{ref}} \frac{P}{{P_{\text{ref}} }}} \right]$$

Equation (12) clarifies that only *α* (*T*) is dependent on the temperature of the model surface. This indicates that the effect of temperature change can be corrected even if the temperature is not uniform over the model. The resulting change in pressure Δ*P* owing to a temperature distribution based on a change in the temperature-correction factor Δ*α*(*T*) can be evaluated by the following equation.

13 $$\frac{\Delta \alpha (T)}{\alpha (T)} = B_{\text{ref}} \cdot \frac{\Delta P}{P}$$

## Experimental setup and conditions

### PSP formulation

We chose PdTFPP as the sensor molecule and poly(TMSP) as the binder. A PSP composed of PdTFPP and poly(TMSP) is known to have good pressure sensitivity under low-pressure conditions (Niimi et al. 2005; Mori et al. 2006), and thereby, is considered to be applicable to the low-pressure experiment in the MWT (Ono et al. 2010). The composition of the PSP is PdTFPP (4.8 mg), poly(TMSP) (0.16 g), and toluene (20 ml). The absorption and emission peaks are approximately 407 and 670 nm, respectively.

### Experimental setup for sample tests

**Fig. 2:**
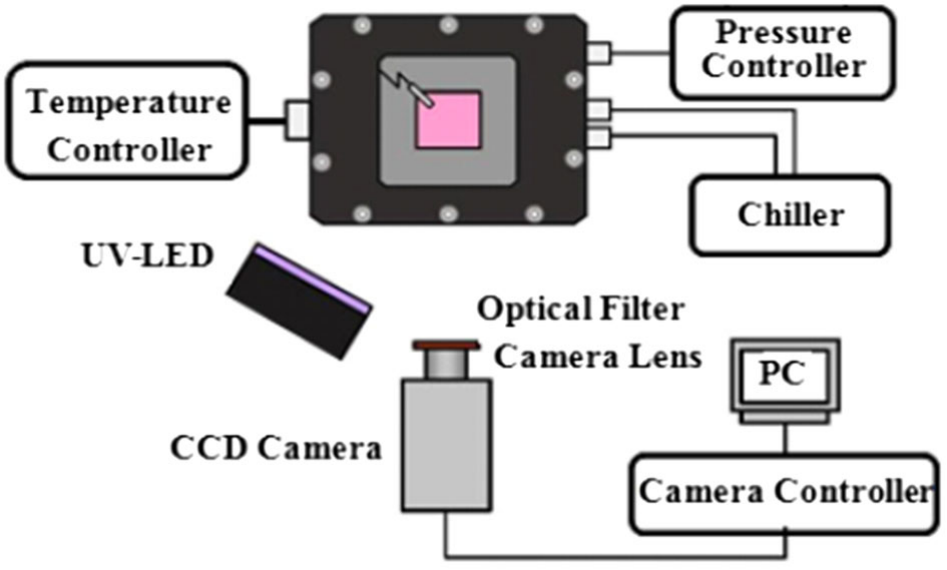
Setup for the calibration tests

The experimental setup for sample tests is illustrated in Fig. 2. A PSP-coated sample plate made of aluminum was placed in a calibration vacuum chamber where the inner pressure and temperature could be controlled from 0.1 to 105 kPa and from ambient temperature to 213 K, respectively. The PSP was excited by a UV-emitting LED unit with a peak wavelength of 395 nm. A band-pass filter (transmission wavelength 400 ± 50 nm) was placed in front of the light source to remove undesirable near-infrared radiation. The luminescence from the PSP sample was captured by a thermoelectrically cooled 12-bit CCD camera (Hamamatsu C4742-95-12ER) with an optical band-pass filter (transmission wavelength 670 ± 20 nm). The film thicknesses of the PSP membranes used for the temperature dependence, photodegradation, and pressure-sensitivity calibration tests were approximately 1.2, 2.2, and 1.2 μm, respectively.

### Wind tunnel and test models

The MWT consists of a vacuum chamber, an induction wind tunnel, and a buffer tank. The induction-type wind tunnel is located inside the vacuum chamber where the pressure of the Martian atmosphere can be simulated. The test gas used typically is dry air, but it can be replaced with carbon dioxide. The wind tunnel is driven by multiple-nozzle supersonic ejectors located downstream of the test section. The gas inside the vacuum chamber is exhausted to the buffer tank through a connecting flexible pipe. The pressure in the vacuum chamber is kept constant by controlling a butterfly valve placed in the connecting pipe. For air-mode operation, the MWT can cover a Reynolds number range from 10^3^ to 10^5^ for airfoil models with the chord length of 50 mm. A Mach number higher than 0.7 can be achieved at a total pressure of 1 kPa. Currently, the MWT is being operated at ambient temperature only.

**Fig. 3:**
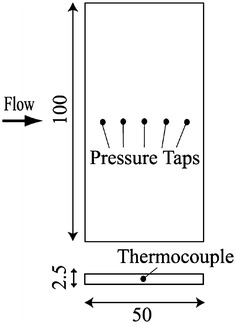
Model coated with the PSP

The test section of the MWT is 100 mm wide and 150 mm high. The upper and lower walls diverge downstream to compensate for the evolution of wall boundary layers. As shown in Fig. 3, an aluminum flat plate with a blunt leading edge was used as a test model. The chord length of the plate was 50 mm, the thickness 2.5 mm, and the span length 100 mm. One thermocouple and five pressure taps were provided on the centerline of the model. The pressure taps were connected to an electric pressure scanner (PSI Model 9116), which has a sufficient response time, through Teflon tubes of 1 mm diameter and about 400 mm length.

### Optical setup for wind tunnel tests

**Fig. 4:**
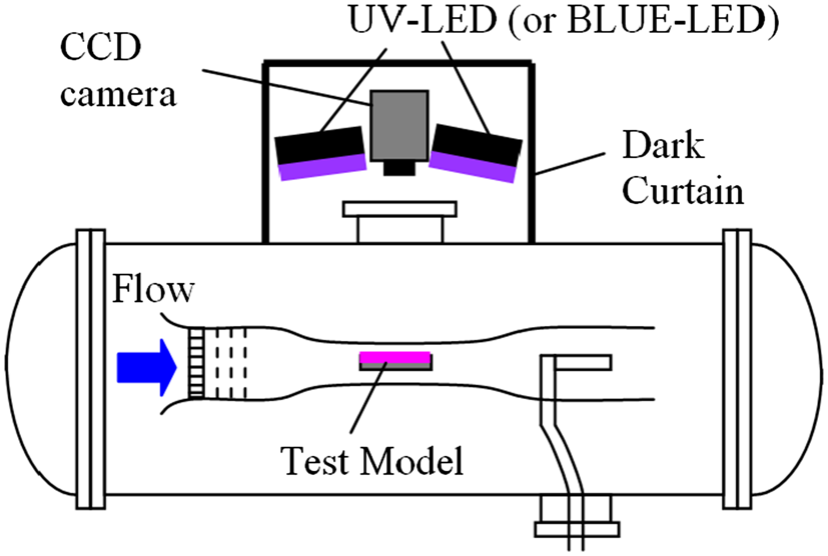
Optical setup for PSP measurements in the MWT

The setup for PSP measurements is shown in Fig. 4. The optical equipment was placed outside the vacuum chamber and measurements were made through an optical window on the top of the vacuum chamber. We used the same LED units and CCD camera as used for the calibration tests. The exposure time and frame rate were set to 359 ms and 3 frames per second, respectively. A F/5.6 camera lens (TAMRON SP23A) and a 560 nm long-pass filter (HOYA O-56) were attached to the camera. The spatial resolution of the PSP measurements was approximately 140 μm. To filter out the background noise, we preliminarily obtained a dark-current image in the wind-off condition, and this image was subtracted from the reference images and the wind-on images.

### Experimental conditions and method

The pressure sensitivity of the sample used for the calibration tests in the calibration chamber was evaluated in the pressure range from 0.4 to 1 kPa and from 1 to 10 kPa using air as a test gas. The temperature was adjusted from 278 to 293 K.

The total pressure in the wind tunnel was varied from 1 to 10 kPa, and the corresponding chord Reynolds number was from 3.2 × 10^4^ to 3.4 × 10^4^. The Mach number was set at around 0.3 (from 0.29 to 0.31) by changing the supply pressure of the ejectors from 0.2 to 0.65 MPa. The angle of attack was fixed at 0 degrees.

## Results and discussions

### Sample calibration tests

#### Sample tests

**Fig. 5:**
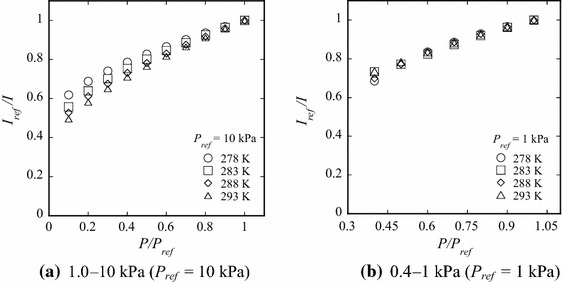
Dependence of pressure sensitivity on temperature

The temperature dependence of the pressure sensitivity of PdTFPP/poly(TMSP) is shown in Fig. 5a for the pressure range 0.4 to 1 kPa and Fig. 5b for 1 to 10 kPa, and the reference pressure (*P*_ref_) of each range considered was 1 and 10 kPa, respectively. The luminescence intensity was obtained by spatially averaging the intensity over all areas of the sample plate. The pressure sensitivity defined from the slope of *I*_ref_ */I* versus *P/P*_ref_, assuming a linear relation, indicates that the pressure sensitivity decreases with decreasing temperature in both Fig. 5a and b, i.e., the luminescence intensity *I* decreases with increasing temperature. This is attributed to an increase in *k*_1_ and *D*_Q_ given by Eqs. (7) and (9), respectively, where both *k*_1_ and *D*_Q_ increase with increasing temperature. Consequently, *k*_D_ and *k*_q_ increases, and the luminescence intensity thereby decreases [see Eq. (1)]. However, Takada et al. (1985) and Masuda (1988) revealed some unique features of poly(TMSP). Their results show that the activation energy for diffusion is nearly zero, and the gas permeability of poly(TMSP) has negligible temperature dependence. Therefore, we deduce that the dominant factor causing the observed decrease in the luminescence intensity is *k*_1_. In addition, the temperature dependence of the pressure sensitivity in the pressure range 1.0 to 10 kPa is large compared with that from 0.4 to 1 kPa. It is considered that this is due to the rate constant *k*_q_, which depends on both the oxygen pressure and the surface temperature (Niimi et al. 2005).

**Fig. 6:**
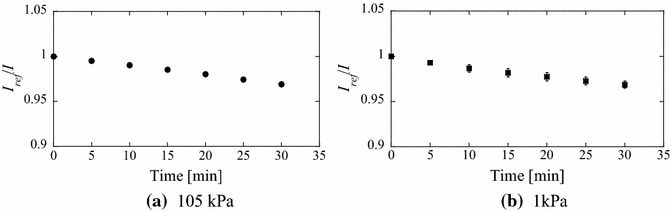
Results of the photodegradation test

Figure 6 shows the effect of photodegradation due to continuous UV light irradiation. This test was repeated three times and each dot indicates an averaged value. Error bars are shown in Fig. 6, although they are negligibly small. The luminescent intensity decreases approximately 3 % over 30 min. Therefore, the effect of photodegradation is considered negligible in MWT tests because the test time is usually no more than 1 min.

**Fig. 7:**
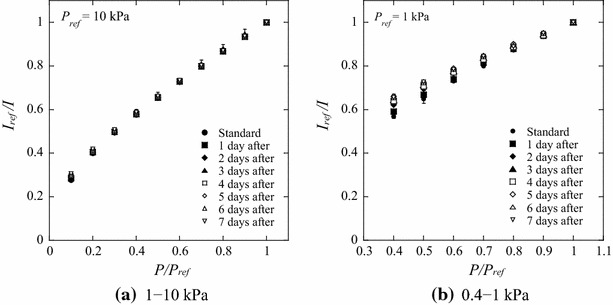
Pressure-sensitivity variation as a factor of time after PSP application to the model

**Fig. 8:**
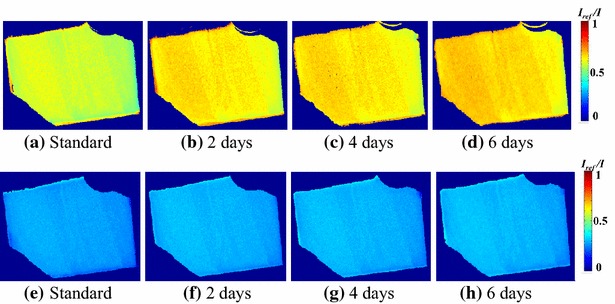
Luminescence-intensity distribution of sample image (**a**–**d**: *P*_ref_ = 1 kPa, with a chamber pressure of 0.4 kPa; **e**–**h**: *P*_ref_ = 10 kPa, with a chamber pressure of 1 kPa)

Figure 7 shows the daily variation of the luminescence intensity. The day when the PSP was applied to the model is regarded as the standard. Little change is observed in the slope of *I*_ref_ */I* versus *P/P*_ref_, corresponding to the pressure sensitivity, in the pressure range 1–10 kPa. However, the pressure sensitivity deteriorates in the pressure range 0.4–1 kPa. The variation over 4 days is notable, whereas little change is observed from the fifth day and later. It should be noted that the reference luminescence intensity *I*_ref_ at the reference pressure *P*_ref_ is different between Fig. 7a and b. Although the pressure sensitivity shown in Fig. 7b decreases 34 % over 7 days, the degradation of the pressure sensitivity becomes relatively large because the luminescence intensity is compared over the narrow pressure range of 0.4–1 kPa. The decrease in the pressure sensitivity with time is due to the variation of the oxygen permeability of poly(TMSP). Masuda et al. (2013) investigated the time dependence of the gas permeability of poly(TMSP). They concluded that the decrease in the gas permeability of poly(TMSP) is caused by both the decrease of microvoids due to structural relaxation and the adsorption of organic vapors. Reduction of the gas permeability of poly(TMSP) was also investigated by Nagai et al. (2001). Their results revealed the effect of physical aging under vacuum conditions using an oil diffusion pump compared with the vacuum conditions using a molecular turbopump. When poly(TMSP) is stored under vacuum using an oil diffusion pump, it absorbs pump oil vapors. Consequently, the decline in oxygen permeability is caused by contamination of the polymer with the hydrocarbon oil. In our calibration test, we did not use an oil diffusion pump but an oil-sealed rotary pump. Hence, we consider that the effect of contamination by pump oil vapors on the oxygen permeability of poly(TMSP) is negligible in the present study. The luminescence-intensity distribution on the sample plate is shown in Fig. 8. The luminescence intensity on the sample plate is nearly uniform, and a spatial nonuniformity due to the absorption of organic vapors is not observed. From these results, we deduce that the observed decrease in the pressure sensitivity shown in Fig. 7b is primarily caused by structural relaxation with time.

#### Calibration in the wind tunnel

**Fig. 9:**
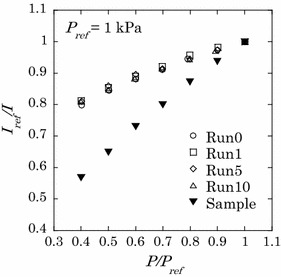
Pressure sensitivity of the sample test and for each wind tunnel test run

**Fig. 10:**
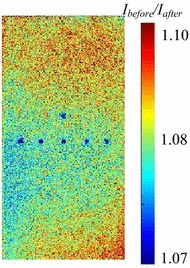
Luminescence intensity distribution without calibration at 1 kPa

Figure 9 shows the pressure sensitivity of the PSP on the flat plate in the pressure range 0.4–1 kPa in the wind-tunnel tests compared with the sample test result. The luminescence intensity in the wind-tunnel tests was obtained by locally averaging the intensity over a 100 × 100 pixel area around the center of the test plate. The calibration tests were repeated 10 times in the wind-off condition by evacuating the air from the wind tunnel chamber and operating the ejector to ventilate the test model in each run, with the exception of Run 0 shown in Fig. 9, which indicates the test before the first ventilation. The pressure sensitivity of PdTFPP/poly(TMSP) in the wind tunnel tests is lower in comparison to the pressure sensitivity in these sample tests. The pressure sensitivity in the wind tunnel is nearly half of the pressure sensitivity in the sample test. An oil-sealed rotary pump was used for the sample tests, whereas an oil diffusion pump was utilized for the wind-tunnel tests. When poly(TMSP) is stored under vacuum conditions using an oil diffusion pump, the pressure sensitivity of the PSP decreases due to the contamination of the polymer with oil vapors (Nagai et al. 2001), as described in “Sample tests”. Hence, the decline in the pressure sensitivity is attributed to contamination of the polymer by oil vapors that derive from the oil diffusion pump. Figure 10 shows the distribution of the luminescence-intensity ratio calculated from the wind-off images taken before and after the tunnel run. The spatially non-uniform distribution, which is not observed in the sample test using the oil-sealed rotary pump, is also due to oil contamination. Dust included in the test gas may also contribute to contamination of the polymer. However, dust contamination had little effect as almost no variation is observed between Run 0 and Run 10, as shown in Fig. 9. From these results, a calibration method that applies the calibration coefficients in the Stern–Volmer relation to the entire image, the typical a priori calibration, cannot be applicable to the MWT tests.

To obtain calibration coefficients using the Stern–Volmer relation, we employed the a priori (direct) model calibration: the PSP-coated model was installed in the test section, and the relation between the luminescence intensity ratio *I*_ref_/*I* and the pressure ratio *P*/*P*_ref_ was obtained by varying the pressure inside the wind-tunnel chamber. The temperature change on the test model during the calibration was about 0.18 K at maximum over the entire pressure range from 1 to 10 kPa. As shown in Fig. 5a, the effect of this temperature change on the pressure sensitivity can be considered negligible.

**Fig. 11:**
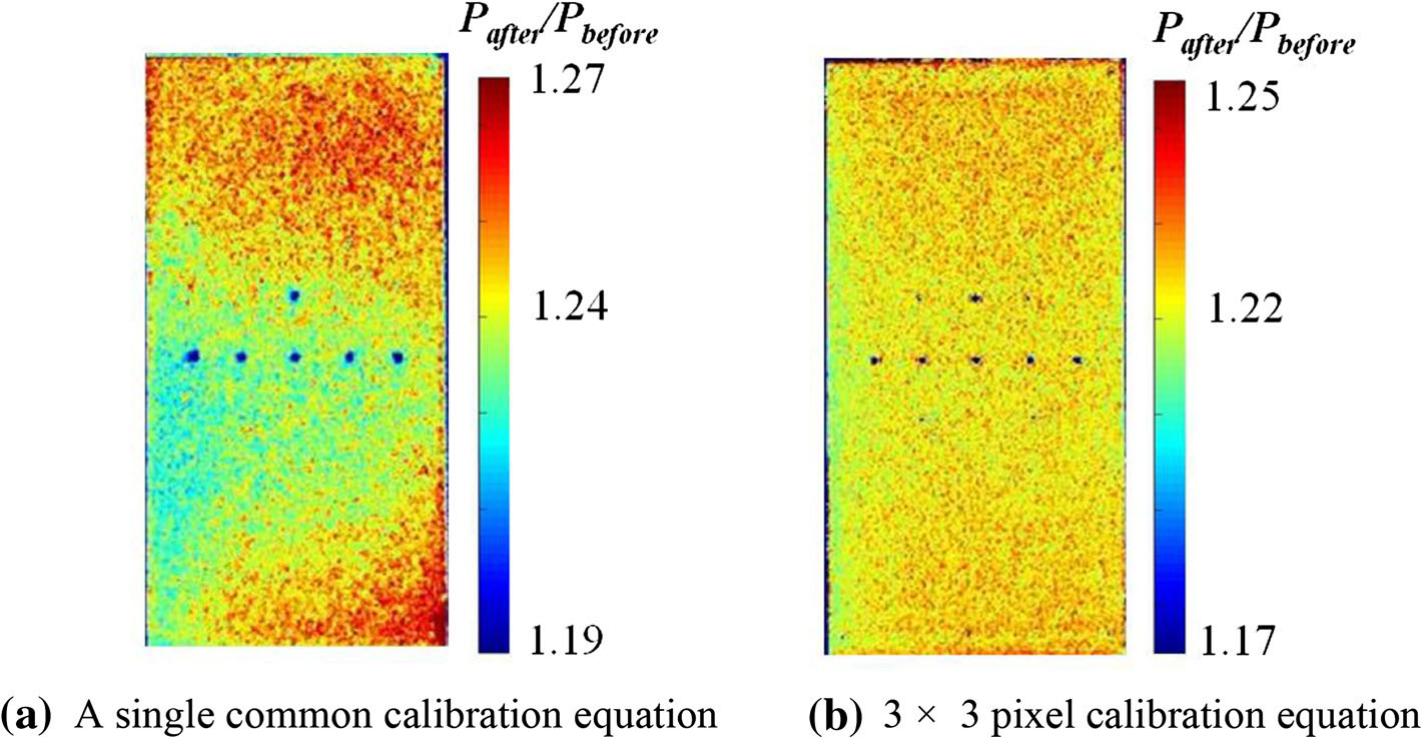
Pressure/ratio distribution in wind-off condition at 1 kPa

In this method, the calibration coefficients are obtained for each pixel of the image of the model using the direct model calibration method. If a common calibration equation is applied to all pixels, the nonuniformity in the pressure ratio *P*_after_ */P*_before_ distribution calculated from the wind-off images taken before and after the tunnel run is observed, as shown in Fig. 11a. In contrast, the nonuniformity can be considerably canceled when the calibration equation obtained at each 3 × 3 pixel area is applied to the appropriate pixel area. Hence, we consider that pixel-based calibration is an appropriate method for the MWT tests. The following results are discussed based on the pixel calibration method.

The temperature change on the test plate during the test time is 0.02 and 1.04 K at chamber pressures of 1 and 10 kPa, respectively. The effects of the temperature changes on the pressure sensitivity are 0.482 and 2.15 %/K at 1 and 10 kPa, respectively, indicating that the variation of the pressure coefficient *C*_p_ due to the temperature change is Δ*C*_p_ = 0.0045 at 1 kPa and Δ*C*_p_ = 0.67 at 10 kPa. The pressure coefficient is defined as the following equation:

14 $$C_{\text{p}} = \frac{{p - p_{\infty } }}{{\frac{1}{2}\rho U^{2} }},$$

where *p* is the local static pressure, *p*_∞_is the main-stream static pressure, *ρ* is the density of the main stream, and *U* is the main-stream velocity. The variation Δ*C*_p_ can be minimized to within 0.12 at the most using the temperature calibration involving *α* (*T*) and the reference images taken immediately after the tunnel shutdown. Furthermore, Δ*C*_p_ eventually reduces to within 0.05 by shifting the PSP pressure level to the pressure measured by the static pressure tube, which is called in situ calibration.

**Fig. 12:**
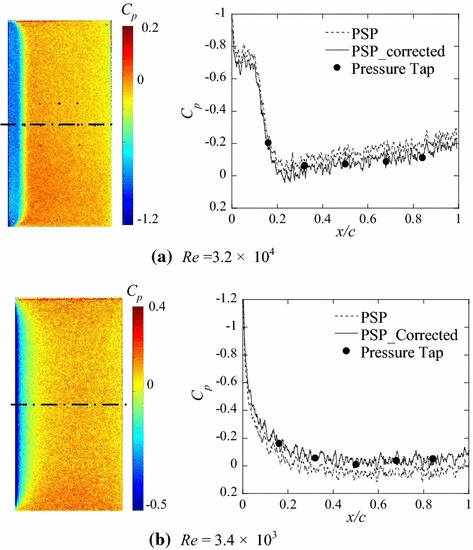
*C*_p_ distribution on the centerline of the test model at an angle of attack of 0° in the wind-on condition

Figure 12 shows wind-on images at the angle of attack of 0° and the *C*_p_ distributions along the centerline of the panel for total pressures of 10 kPa (*Re* = 3.2 × 10^4^) in (a) and 1 kPa (*Re* = 3.4 × 10^3^) in (b). The wind-on image was obtained by averaging eight images and was normalized by the wind-off image. The pressure distribution results with and without both temperature correction and in situ calibration are compared in the right-hand graphs of Fig. 12a, b, for total pressures of 10 kPa (*Re* = 3.2 × 10^4^) and 1 kPa (*Re* = 3.4 × 10^3^), respectively.

In such a low-Reynolds-number flow around a flat plate with a blunt leading edge, flow separates at the leading edge and reattaches to the surface after transition. Then, a laminar separation bubble forms between the separation and reattachment locations (Ota 1976). The negative pressure regions around the leading edge observed in Fig. 12 indicate the presence of the laminar separation bubble. The transition location can be identified as the point where the surface pressure begins to rapidly recover (Tani 1964). O’Meara and Mueller (1987) suggested that the reattachment location can be defined as the location downstream of the transition point where a rapid decrease in the rate of the pressure recovery is observed. On the basis of this approach, Gerakopulos et al. (2010) proposed that the reattachment point was estimated as the location of the local minimum in the second derivative of the polynomial fit. In accordance with these criteria, the transition point can be estimated at around *x/c* = 0.1 at *Re* = 3.2 × 10^4^. However, no rapid increase in the *C*_p_ distribution at *Re* = 3.4 × 10^2^ is observed in Fig. 12b, indicating that the separated shear layer does not undergo a laminar-to-turbulent transition. The reattachment locations are estimated at approximately *x/c* = 0.22 at *Re* = 3.2 × 10^4^ and *x/c* = 0.21 at *Re* = 3.4 × 10^3^.

Tani et al. (1961) conducted surface pressure measurements for the flow over a backward-facing step. Their results showed that the separated flow at the corner does not undertake a transition and reattach in a laminar state when the step height is very small, and that the pressure on the surface slowly recovers without a steep pressure recovery due to a laminar-to-turbulent transition. The *C*_p_ distribution shown in Fig. 12b reveals a feature very similar to the moderate pressure recovery in the step flow observed by Tani et al. Sasaki and Kiya (1991) investigated a separation bubble on a flat plate using a water tunnel and concluded that the separated shear layer is still laminar at the reattachment point based on visualization results over 2.0 × 10^3^ < *Re* < 6.0 × 10^3^. These past results support our results and explanation, and it can be said that this PSP measurement technique can quantitatively and qualitatively capture fluid physics phenomena at low Reynolds numbers under low-pressure conditions.

## Conclusion

A pressure-sensitive paint technique has been developed for surface pressure measurements at low Reynolds number in the Mars wind tunnel at Tohoku University. Sample tests were conducted to evaluate the characteristics of the PSP. A blunt, flat-plate model was tested in the MWT to assess the effectiveness of the PSP technique. A PSP consisting of PdTFPP and poly(TMSP) exhibits high-pressure sensitivity at low pressures and is suitable for use in low Reynolds number testing in the MWT. It was determined that the pressure sensitivity of the PSP is not uniform over the model surface and decreases gradually with time. These factors can be canceled by conducting a pixel-by-pixel calibration in the tunnel. The accuracy of PSP measurements can be improved by using the wind-off image obtained just after the tunnel run and by applying a temperature correction. The PSP data are in good quantitative agreement with pressure tap data, and we succeeded in quantitatively and qualitatively measuring characteristic fluid phenomena at low Reynolds numbers such as a leading edge separation bubble.
